# Supplementary material for: FruitFire: a luciferase based on a fruit fly metabolic enzyme
Source: bioRxiv. 2023 Jun 30:2023.06.30.547126. Preprint. [Version 1] doi: 10.1101/2023.06.30.547126 (PMC10327219; doi:10.1101/2023.06.30.547126)
Supplement: Supplement 1 [file media-1.pdf]

Supporting Information for

FruitFire: a luciferase based on a fruit fly metabolic enzyme

Spencer T. Adams, Jr., Jacqueto Zephyr, Markus F. Bohn, Celia Schiffer and Stephen C. Miller*

Department of Biochemistry and Molecular Biotechnology, UMass Chan Medical School, 364 Plantation St, Worcester, MA 01605

Stephen.miller@umassmed.edu

Table of Contents:

Supplementary Figures 1-6 S2-7

Supplementary Table 1 S8

A

Mutant #	ASGG (312-315)	F → L (285)	TTSA (344-347)	LCGG (312-315)	TETTSA (342-347)	A → G (245)	T → R (219)
1	•	•					
2	•						
3	•		•				
4				•			
5					•	•	•
6					•	•	
7					•		
8	•	•			•	•	•
9	•	•			•	•	
10	•				•	•	•
11	•	•			•		•

B

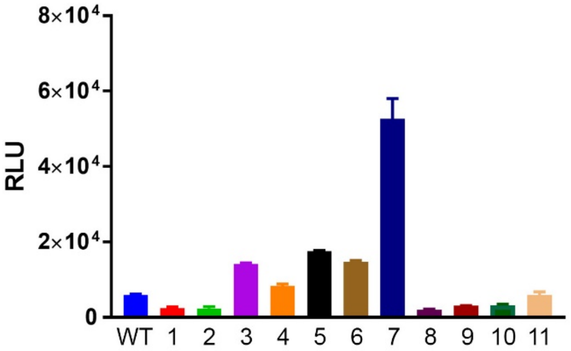

C

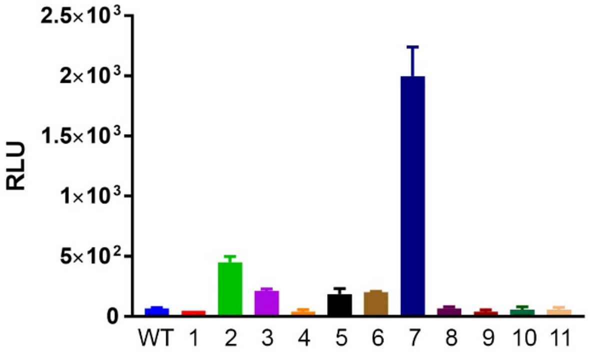

Supplementary Figure S1. ***In vitro*** **activity of CG6178 active site mutants with CycLuc2 and D-luciferin.** A) Residues in CG6178 highlighted in Figure 2 were replaced with the corresponding Fluc sequence in various combinations as shown. CG6178 and mutants 1-11 (40 nM) were mixed with 250 μM of B) CycLuc2 or C) D-luciferin and light emission was measured with a Promega GloMax.

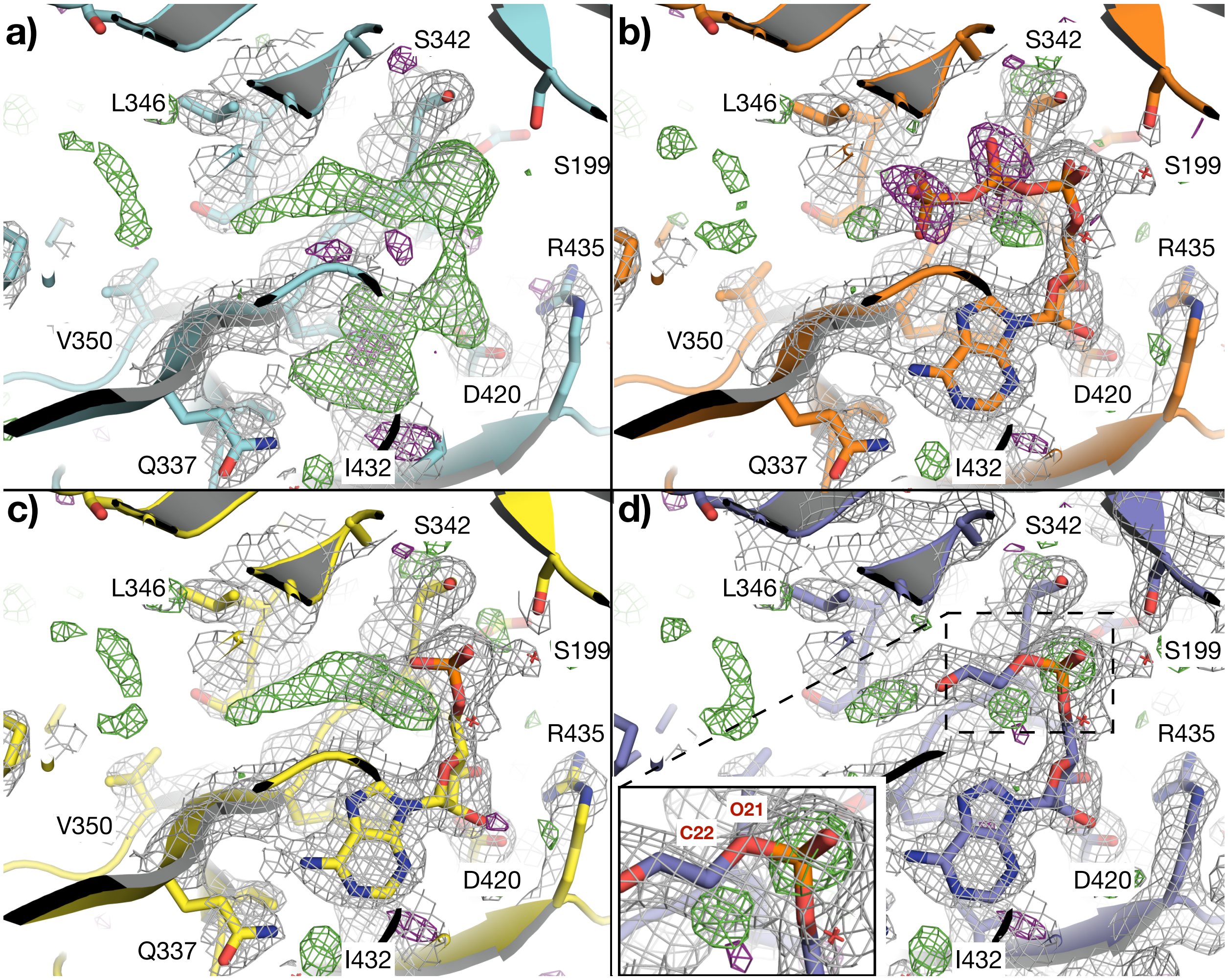

Supplementary Figure S2: **ATP, AMP, and an adenylated ethylene glycol are insufficient to satisfy the density in the active site of CG6178**. (**a**) The active site of CG6178 with no ligand modeled. (**b**) ATP, (**c**) AMP, and (**d**) an adenylated ethylene glycol molecule modeled in the ligand electron density. CG6178 is shown in cartoon representation. The 2Fo-Fc map (grey, at σ = 1) and Fo-Fc difference maps (green σ = +3.0 and purple σ = -3.0) are shown.

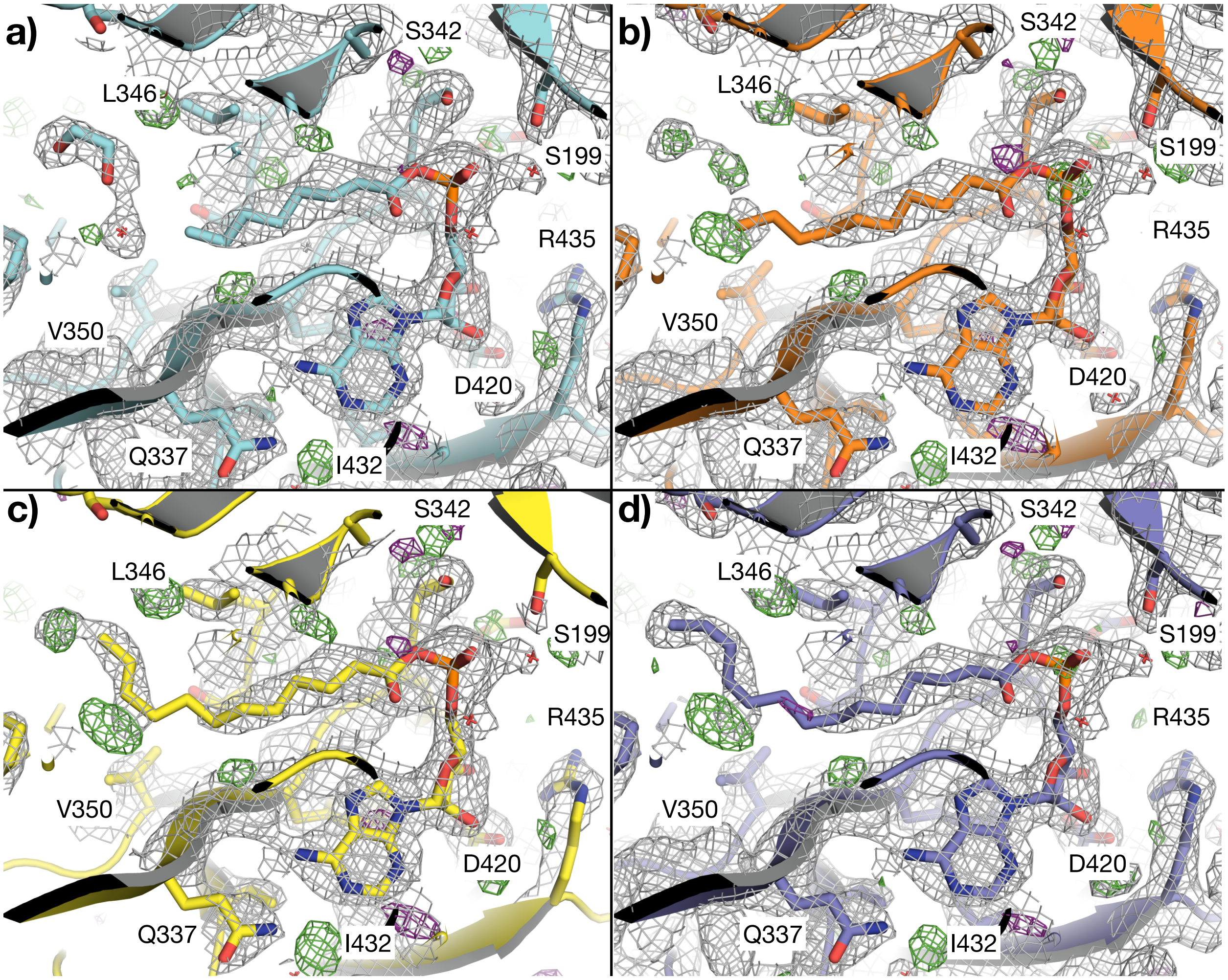

Supplementary Figure S3: **Octanoyl-AMP (C8) represents the best model for the ligand density.** (**a**) Octanoyl (C8), (**b**) Decanoyl (C10), (**c**) Dodecanoyl (C12), and (**d**) Myristoyl (C14) AMP esters modeled in the active site of CG6178. CG6178 is shown in cartoon representation. The 2Fo-Fc map (grey, at σ = 1) and Fo-Fc difference maps (green σ = +3.0 and purple σ = -3.0) are shown.

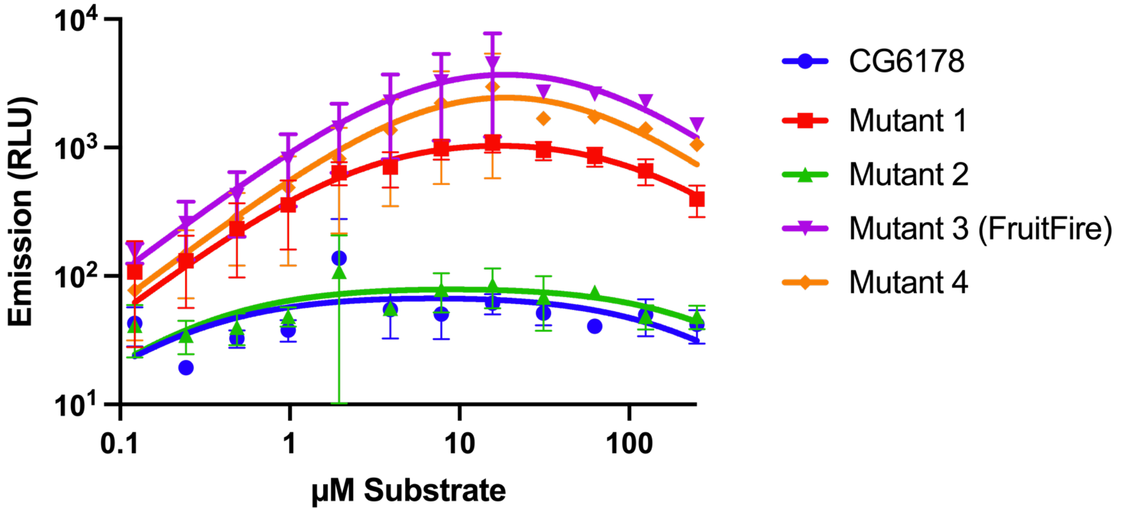

Supplementary Figure S4. ***In vitro*** **performance of CG6178 and mutants with D-luciferin (0.122 - 250 μM).** Signal from CG6178 and Mutant 2 is not above background.

A

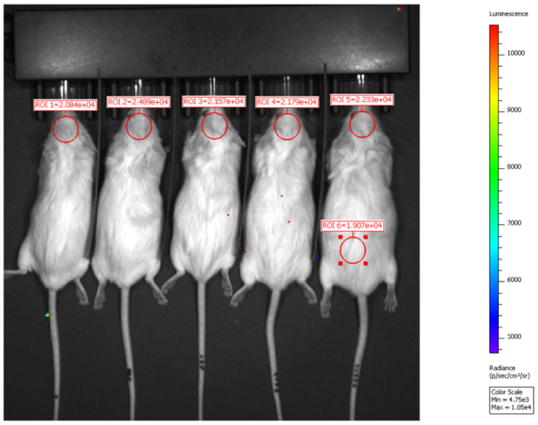

B

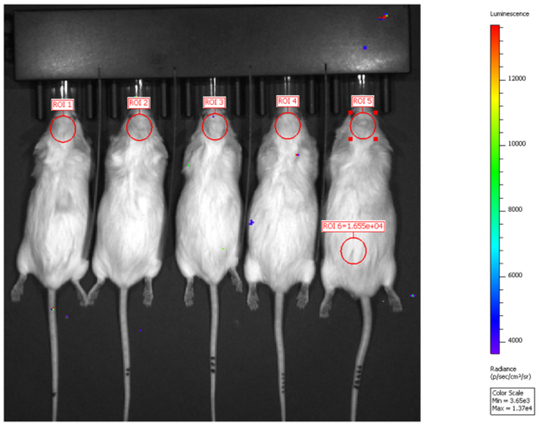

C

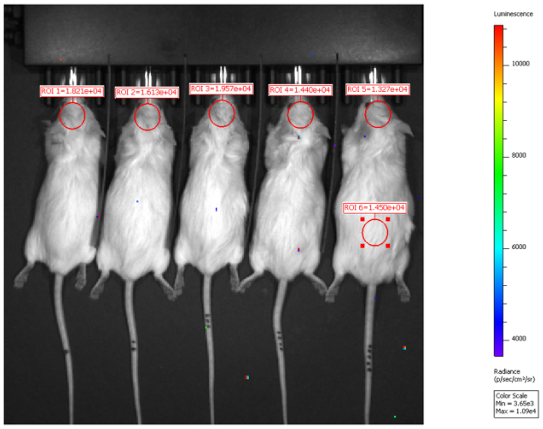

Supplementary Figure S5. Imaging of mice transduced in the striatum with CG6178. Mice were imaged after ip injection with A) 0.1 mL of 100 mM D-luciferin, B) 0.1 mL of 2.5 mM CycLuc2, or C) 0.1 mL of 250 µM CycLuc2-amide.

A

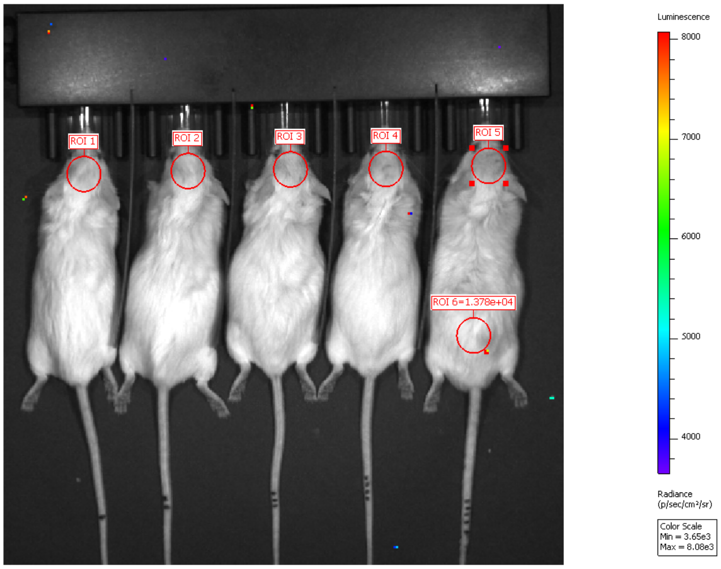

B

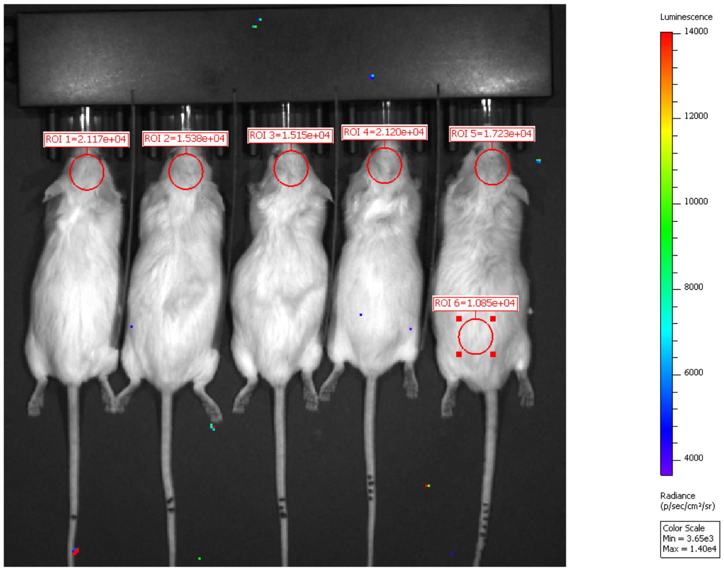

Supplementary Figure S6. Imaging of mice transduced in the striatum with FruitFire. Mice were imaged after ip injection with A) 0.1 mL of 100 mM D-luciferin or B) 0.1 mL of 2.5 mM CycLuc2.

Table S1. Data collection and refinement statistics for CG6178.

**Data Collection**	
**Wavelength**	1.033
**Resolution range**	30.72 - 2.5 (2.589 - 2.5)
**Space group**	P 21 21 21
**Unit cell**	55.851 77.778 113.315 90 90 90
**Total reflections**	99870
**Unique reflections**	17673 (1726)
**Multiplicity**	5.6
**Completeness (%)**	99.80 (99.37)
**Mean I/sigma(I)**	17.46 (2.95)
**Wilson B-factor**	36.31
**R-merge**	0.104 (0.671)
**Refinement**	
**Reflections used in refinement**	17660 (1724)
**Reflections used for R-free**	885 (87)
**R-work**	0.1949 (0.2515)
**R-free**	0.2527 (0.3272)
**Number of non-hydrogen atoms**	4123
**macromolecules**	3988
**ligands**	69
**solvent**	99
**Protein residues**	534
**RMS(bonds)**	0.002
**RMS(angles)**	0.50
**Ramachandran favored (%)**	95.53
**Ramachandran allowed (%)**	4.72
**Ramachandran outliers (%)**	0.75
**Rotamer outliers (%)**	1.67
**Clashscore**	15.16
**Average B-factor**	38.72
**macromolecules**	38.72
**ligands**	42.50
**solvent**	37.28

Statistics for the highest-resolution shell are shown in parentheses.

[b]*R-merge*= $\Sigma \mid I - \langle I \rangle \mid / \Sigma I$, where I = observed intensity, $\langle I \rangle$ = average intensity over symmetry equivalent; values in parentheses are for the highest resolution shell.

[c]RMSD, root mean square deviation.

[d]*R-work* = $\Sigma \| F_o| - |F_c\| / \Sigma |F_o|$.

[e]*R-free* was calculated from 5% of reflections, chosen randomly, which were omitted from the refinement process
